# Lingual Thyroid Unmasked by Acute Stroke: A Hidden Airway Emergency

**DOI:** 10.7759/cureus.99162

**Published:** 2025-12-13

**Authors:** Yuichiro Yoneoka, Akane Tange, Kohei Honda, Nobumasa Ohara

**Affiliations:** 1 Department of Neurosurgery, Uonuma Kikan Hospital, Minami-Uonuma, JPN; 2 Department of Endocrinology, Uonuma Kikan Hospital, Minami-Uonuma, JPN; 3 Department of General Internal Medicine, Uonuma Kikan Hospital, Minami-Uonuma, JPN; 4 Department of Otorhinolaryngology, Uonuma Kikan Hospital, Minami-Uonuma, JPN

**Keywords:** airway obstruction, ectopic thyroid, ischemic stroke, lingual thyroid, stroke-related bulbar dysfunction, tracheostomy

## Abstract

Ectopic thyroid tissue, most commonly presenting as a lingual thyroid, is a rare congenital anomaly that is typically asymptomatic. However, when symptomatic, it can cause local symptoms, such as dysphagia, or, rarely, acute airway compromise. We report a unique case where a previously compensated lingual thyroid became immediately life-threatening due to acute stroke-related bulbar motor dysfunction.

A 63-year-old man presented with an acute unilateral stroke (left corona radiata infarct). Emergency noncontrast head CT, performed according to the stroke protocol, incidentally revealed a large, 4.0 cm, calcified mass at the tongue base, severely narrowing the oropharyngeal airway. Acute stroke-related bulbar motor dysfunction (manifested as dysarthria and impaired pharyngeal control) compromised the patient's airway protective reflexes, creating a high risk of obstruction and aspiration. Iodine-123 scintigraphy confirmed that the mass was the patient’s sole functioning thyroid tissue (absent orthotopic gland). An airway-first multidisciplinary strategy was immediately initiated: tracheostomy under local anesthesia, followed by definitive *en bloc* resection via midline mandibulotomy/glossotomy with vascular control. The patient was successfully transitioned to lifelong thyroid hormone replacement and achieved a good functional recovery.

This case demonstrates that even mild, unilateral stroke-related bulbar motor dysfunction can be sufficient to unmask a previously compensated anatomical obstruction (lingual thyroid), resulting in an acute airway emergency. Effective management requires an immediate, multidisciplinary approach centered on securing the airway safely before tumor resection.

## Introduction

Ectopic thyroid tissue is a rare congenital anomaly, and lingual thyroid accounts for up to 90% of all ectopic thyroid cases [1]. Although congenital, the condition is frequently asymptomatic until adulthood. When symptomatic, lingual thyroid commonly presents in the second to fourth decades of life with dysphagia, dysphonia, foreign-body sensation, or obstructive symptoms, often precipitated by hormonal changes (puberty, pregnancy) or chronic elevation of thyroid-stimulating hormone (TSH), which promotes glandular hypertrophy [1-3].

Management strategies range from non-surgical options (TSH suppression, radioablation) to surgical approaches, including transoral techniques (laser, robotic surgery) for smaller lesions and external approaches (transhyoid, suprahyoid, mandibulotomy) for larger or highly vascular lesions requiring superior exposure and vascular control [4]. Acute airway compromise from a lingual thyroid is rare and typically requires a secondary precipitating factor that disrupts pharyngeal compensation mechanisms.

We present a case in which a giant lingual thyroid remained silent until the patient suffered an acute unilateral corona radiata infarct, which impaired bulbar motor function and unmasked a previously compensated airway obstruction.

## Case presentation

**Figure 1:**
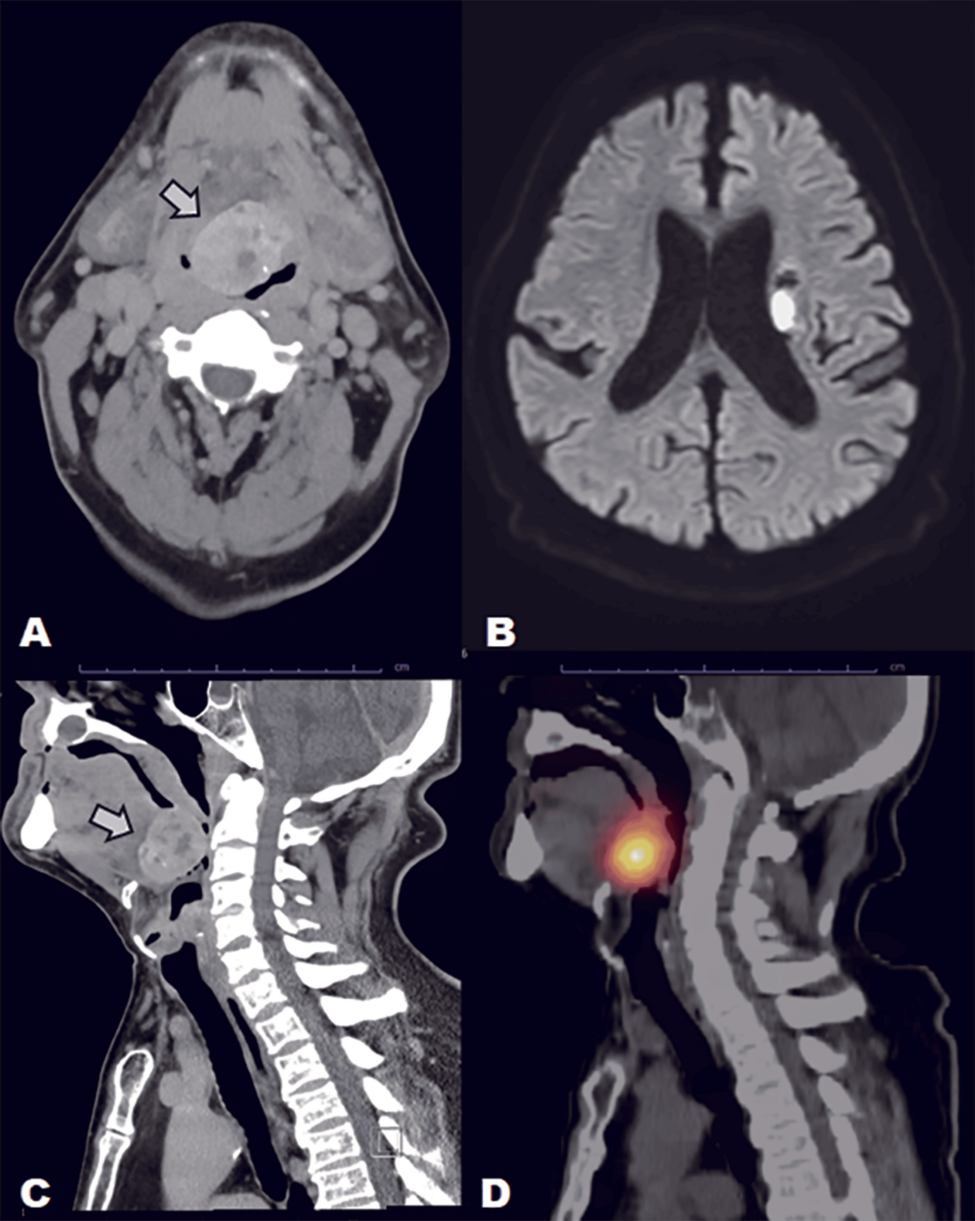
Preoperative multimodality imaging A. Axial noncontrast CT showing a 4.0 × 3.5 × 3.0 cm calcified tongue-base mass causing severe oropharyngeal airway narrowing; B. Axial DWI revealing acute infarction in the left corona radiata; C. Sagittal CT MPR demonstrating marked airway compression by the mass; D. I-123 scintigraphy showing intense uptake at the tongue base with absent cervical activity, confirming ectopic lingual thyroid

A 63-year-old man presented to our emergency department 5 days after the onset of right hemiparesis and dysarthria (National Institutes of Health Stroke Scale (NIHSS) 13). He had delayed seeking medical attention due to his aversion to hospitals. Noncontrast head computed tomography (CT) excluded hemorrhage but revealed a tongue-base mass measuring 4.0 × 3.5 × 3.0 cm with coarse calcification, causing significant narrowing of the oropharyngeal airway (Figure 1A).

Magnetic resonance imaging (MRI) confirmed an acute left corona radiata infarct (Figure 1B). Sagittal multiplanar reconstruction (MPR) further demonstrated marked airway compromise by the mass (Figure 1C). Thyroid studies showed elevated TSH (11.4 μIU/mL) and low-normal free thyroxine (FT4) (0.99 ng/dL). Iodine-123 (I-123) scintigraphy demonstrated intense uptake at the tongue base with no cervical thyroid activity (Figure 1D), confirming lingual thyroid as the sole functioning thyroid tissue [2].

**Figure 2:**
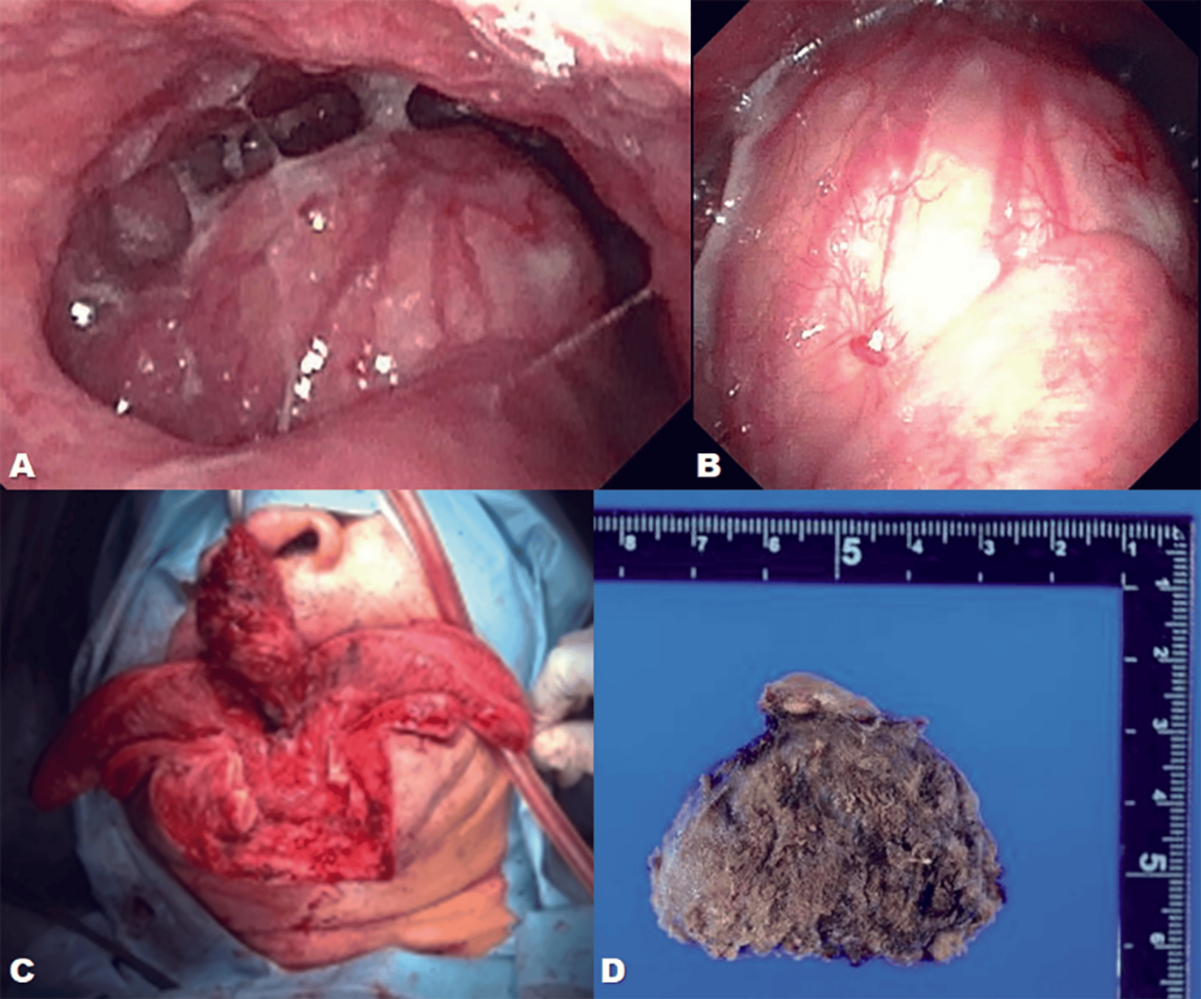
Endoscopic view, intraoperative findings, and gross pathology A. Fiberoptic laryngoscopy showing a protruding tongue-base mass occupying the vallecular space; B. Close view of the mass surface demonstrating smooth mucosal covering; C. Intraoperative view during midline mandibulotomy/glossotomy showing exposure and *en bloc* excision of the mass; D. Gross pathology specimen, cut surface showing fibrosis and calcification consistent with ectopic thyroid tissue

Before definitive intervention, fiberoptic laryngoscopy (Figure 2A and Figure 2B) confirmed a protruding tongue-base mass occupying the vallecular space and compromising the laryngeal inlet. Because acute stroke-related bulbar motor dysfunction (manifested as dysarthria and impaired pharyngeal motor control) created an immediate risk of precipitous airway collapse, an airway-first surgical plan was chosen. Tracheostomy was performed under local anesthesia, followed by midline mandibulotomy and glossotomy for en bloc excision (Figure 2C). Gross pathology revealed a well-encapsulated lesion with fibrosis and calcification (Figure 2D), consistent with ectopic thyroid tissue.

**Figure 3:**
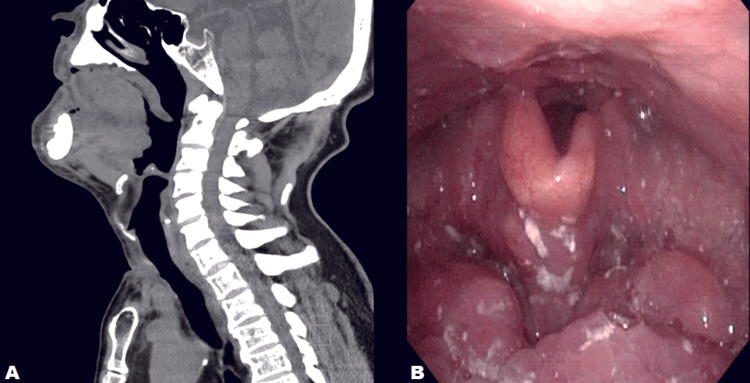
Postoperative airway evaluation A. Postoperative sagittal CT MPR showing complete resolution of oropharyngeal airway obstruction; B. Fiberoptic laryngoscopy after surgery demonstrating a clear, unobstructed laryngeal inlet with restored epiglottic mobility. MPR: multiplanar reconstruction

Postoperatively, aspirin was resumed on day 1. Levothyroxine (125 µg/day) was initiated on day 22 due to the absence of an orthotopic gland, and thyroid indices normalized quickly. Follow-up sagittal MPR demonstrated complete resolution of the oropharyngeal airway obstruction (Figure 3A), and fiberoptic examination confirmed restoration of the laryngeal inlet with improved epiglottic mobility (Figure 3B). The patient transitioned to oral intake and was transferred for rehabilitation.

## Discussion

This case presents a unique and clinically crucial scenario where a common neurological event (acute ischemic stroke) intersected with a rare anatomical anomaly (lingual thyroid) to create an immediate, life-threatening crisis. Our management highlights three critical clinical lessons relevant to internal medicine, endocrinology, and surgery.

The interplay of TSH, compensatory hypertrophy, and airway crisis

The patient presented with subclinical hypothyroidism, a finding consistent with reports that the majority of lingual thyroid cases exhibit functional deficits [3]. The elevated TSH level (11.4 μIU/mL) represents a long-standing compensatory mechanism aiming to maintain euthyroidism. However, this chronic TSH stimulation is the primary driver of the compensatory hypertrophy that ultimately enlarged the mass to 4 cm [3]. The subsequent acute stroke-related bulbar motor deficit then precipitated pharyngeal tone loss, which, combined with the pre-existing, compensated anatomical mass, led to the immediate and critical obstructive/aspiration risk. This mandates that endocrinologists and internists consider the anatomical consequences of chronic TSH stimulation, even when FT4 is maintained within the normal range.

Systematic oropharyngeal assessment on emergency stroke CT

Emergency stroke protocols typically prioritize the brain parenchyma and vasculature. However, this case emphatically argues that the oropharyngeal corridor must be systematically evaluated. The incidental finding of the mass on routine noncontrast head CT (Figure 1A) was the only timely warning sign. Had the mass been missed, the predicted difficulty of intubation and the high risk of aspiration due to stroke-related swallowing dysfunction could have resulted in a catastrophic outcome. This reinforces that stroke imaging must include deliberate assessment of the surrounding anatomy for incidental lesions that become clinically paramount when bulbar function is compromised.

The necessity of the airway-first multidisciplinary strategy

Multiple surgical techniques exist for lingual thyroid resection. Transoral approaches, including transoral robotic surgery (TORS) and direct laryngoscopy with laser, are suitable for smaller (<2 cm) and more accessible lesions. Radioiodine ablation is a non-surgical alternative when the mass is small and the patient is not a good surgical candidate. However, our patient's clinical situation necessitated the most aggressive external approach. Given the size of the mass (4.0 cm), significant vascularity, and the patient’s acute bulbar motor deficit, standard endotracheal intubation carried an unacceptably high risk of failure or tumor manipulation-related obstruction. Our decision to perform a tracheostomy under local anesthesia before proceeding to definitive resection (Figure 2C) was crucial [4]. This airway-first surgical approach prioritized patient safety by securing a definitive airway outside the compromised field, minimizing the risk of perioperative aspiration and acute airway obstruction. Furthermore, multidisciplinary collaboration was essential: Endocrinology for the initial diagnosis and lifelong levothyroxine planning (mandated by the absence of an orthotopic gland [2]), Otorhinolaryngology for the complex airway management and resection, and Neurosurgery/Neurology for the acute stroke management.

Learning points

1. Systematic oropharyngeal inspection on emergency stroke CT is essential, as lesions that are silent under normal conditions may become life-threatening when stroke-related bulbar dysfunction develops.

2. Verification of orthotopic thyroid status is mandatory before resecting a suspected lingual thyroid, as removal of the sole functioning gland necessitates lifelong hormone replacement.

3. An airway-first surgical approach (tracheostomy, followed by mandibulotomy/glossotomy with vascular control) provides the safest pathway for managing large tongue-base lesions that threaten the airway.

## Conclusions

Acute stroke with bulbar motor impairment can unmask previously silent anatomical lesions, converting compensated abnormalities into life-threatening emergencies. Careful oropharyngeal inspection during emergency stroke imaging, verification of orthotopic thyroid status prior to resection, and an airway-first surgical strategy are critical for safe and effective management of such complex cases.
